# High levels of circulating leukocyte microparticles are associated with better outcome in acute respiratory distress syndrome

**DOI:** 10.1186/cc9978

**Published:** 2011-01-18

**Authors:** Christophe Guervilly, Romaric Lacroix, Jean-Marie Forel, Antoine Roch, Laurence Camoin-Jau, Laurent Papazian, Françoise Dignat-George

**Affiliations:** 1 Réanimation Médicale-Détresses Respiratoires Aigües-Infections Sévères, URMITE CNRS-UMR 6236, Hôpital Nord, Assistance Publique, Hôpitaux de Marseille, Chemin des Bourrely, 13015 Marseille, France; 2 UMR-S 608 Inserm, laboratoire d'hématologie et d'immunologie, UFR de pharmacie, université de la Méditerranée, 27, boulevard Jean-Moulin, 13385 Marseille cedex 5, France

## Abstract

**Introduction:**

The current study has addressed the presence and the cellular origin of microparticles (MP) isolated from bronchoalveolar lavage (BAL) fluid and from blood samples from patients with acute respiratory distress syndrome (ARDS). Their prognostic interest was also investigated.

**Methods:**

Fifty-two patients were included within the first 24 hours of ARDS. They were compared to spontaneous breathing (SB) and ventilated control (VC) groups. Bronchoalveolar lavage (BAL) and blood samples were obtained on Day 1 and Day 3 in an ARDS group. Leukocyte microparticles (LeuMP), neutrophil microparticles (NeuMP), endothelial microparticles (EMP), and platelet microparticles (PMP) were measured in arterial blood and in BAL samples by flow cytometry. Mortality from all causes was recorded at Day 28.

**Results:**

All MP subpopulations were detected in BAL. However, only LeuMP and NeuMP were elevated in ARDS patients compared to the SB group (*P *= 0.002 for both). Among ARDS patients, higher levels of LeuMP were detected in blood (Day 1) and in BAL (Day 3) in survivors as compared with the non survivors. Circulating LeuMP >60 elements/microliter detectable on Day 1 of ARDS, was associated with a higher survival rate (odds ratio, 5.26; 95% confidence interval, 1.10 to 24.99; *P *= 0.037).

**Conclusions:**

The identification of the cellular origin of microparticles at the onset of ARDS has identified LeuMP as a biomarker of prognostic significance. The higher levels of LeuMP in survivors could be associated with a protective role of this MP subpopulation. This hypothesis needs further investigations.

## Introduction

Acute Lung Injury (ALI) and its most severe clinical presentation, Acute Respiratory Distress Syndrome (ARDS), occur after a variety of insults, including sepsis, trauma, or aspiration of gastric contents. Despite recent therapeutic advances in the field of mechanical ventilation, 30% to 50% of ARDS patients die [1]. A recent systematic review suggested that mortality from ARDS has not decreased substantially since the publication of the American-European consensus conference in 1994 [2,3].

Converging evidence from clinical and experimental studies shows that leukocytes play a pivotal role in injury during the acute phase of ALI/ARDS. Early in the course of ARDS, lung biopsies and bronchoalveolar-lavage fluid (BAL) show a marked accumulation of neutrophils. Neutrophils are the corner stone of host defenses by releasing proinflammatory cytokines and chemokines that could explain, at least in part, why anti-inflammatory therapies have largely been unsuccessful in ARDS [4].

The local and systemic proinflammatory responses accompanying ARDS are orchestrated by the interactions between circulating cells such as leukocytes, platelets, and endothelial cells. It is now well admitted that during inflammatory responses, cells release submicron vesicles that bud off from the cell membrane. These cell-derived microparticles (MPs) have proven to be sensitive markers for assessing the activation/apoptotic status of cells in many inflammatory disorders such as SIRS, meningococcal sepsis, severe trauma, and neuropaludism [5-7]. Microparticles are submicron plasma membrane vesicles that express cell surface proteins of the original cells and negatively charged phospholipids such as phosphatidylserine. Moreover, MPs behave as vectors of bioactive molecules, which accounts for their pro-coagulant and pro-adhesive potential. Taken together, the interest in MPs has substantially increased, not only for their clinical relevance as disease markers but also for their role as effectors in the tight tuning of adaptive responses such as inflammation, immunity, or hemostasis [8].

The critical role of leukocyte-mediated responses led us to hypothesize that the strong local and systemic inflammatory responses associated with ARDS may be associated with altered levels of leukocyte MPs (LeuMP). The objective of the present study was therefore to measure LeuMP and other MP subpopulations both in blood and BAL early in the course of ARDS. Because recent evidence suggests that poor outcome in critically ill patient is associated with "immune paralysis" (endogenous immunosuppresion) [9], we also postulated that high levels of LeuMP may be associated with better outcome during ARDS.

## Materials and methods

### Inclusion criteria

Patients admitted to the medical ICU of Sainte Marguerite University Hospital were screened daily during a two-year period for enrolment if they met the American-European Consensus Conference (AECC) criteria for ARDS [2]. Patients were included after written informed consent was obtained from each patient's next of kin and after approval by the local ethics committee (comité consultatif pour la protection et la recherche biomédicale de Marseille 1). All subjects were included within the first 24 hours of ARDS. Exclusion criteria included age <18 years, pregnancy, and left ventricular failure. Additionally, we excluded patients with conditions known to be associated with increased circulating levels of MPs such as acute coronary syndromes, severe chronic renal failure (defined as creatinine clearance <30 mL/minute), heparin-induced thrombocytopenia, antiphospholipid syndrome, sickle-cell disease, organ or bone marrow transplantation, neutropenia, and hematological malignancies. Controls consisted of two groups of patients, one with ICU patients mechanically-ventilated for non-pulmonary disorders and the other group included spontaneously breathing subjects who underwent a bronchoscopic procedure as part of a planned work-up for a suspicion of a non-infectious pulmonary disease (chronic cough, *n *= 5, esophageal cancer, *n *= 3, suspicion of sarcoidosis, *n *= 2 and hemoptysis, *n *= 2).

### Data collection

The following demographic data were collected at admission in the ICU: age, gender, cause of ARDS, Simplified Acute Physiology Score II (SAPS II) [10]. The following clinical severity scores were assessed at inclusion in the study: lung injury severity score (LISS) [11] and Sequential Organ Failure Assessment (SOFA) [12]. The presence of an associated septic shock [13] was recorded at inclusion. Ventilator free days were evaluated at 28 days (VFD_28_) for MPs levels comparisons. The following respiratory and hemodynamic variables were assessed within the first 24 hours of ARDS: PaO_2_/FiO_2 _ratio, PaCO_2_, arterial pH, total PEEP, plateau pressure, quasi-static compliance, minute ventilation, tidal volume, heart rate, mean arterial pressure, vasopressor requirements. Standard biological parameters were obtained at inclusion: total leukocyte count, hematocrit, platelet count, prothrombin time, fibrinogen, arterial lactate levels, serum creatinine, and procalcitonin. Neutrophil counts were also performed in BAL. As an index of protein permeability, we have measured the BALF to plasma total protein ratio according to the method of the dilution of urea described by Rennard *et al. *[14].

### Pulmonary and systemic microparticle collections

Bronchoalveolar lavage (BAL) and blood samples were obtained at Day 1 of ARDS and were repeated at Day 3. Arterial blood samples (6 mL) were obtained (from an indwelling arterial catheter) in citrated tubes just before BAL was performed. Directed BAL under fiberoptic bronchoscopy was conducted as previously described [15]. The cells were counted, and the BAL was immediately treated using serial centrifugations (1,500 g for 30 minutes; 13,000 g for 2 minutes). Platelet-free plasma (PFP) samples were prepared, and supernatants were aliquoted and stored at -80°C until analysis.

### Cytometry analysis

Antibodies anti-CD41-FITC (clone PL2-49) and IgG1-FITC (clone 2H11/2H12) were from BioCytex (Marseille, France). Antibodies anti-CD31-PE (clone 1F11), anti-CD45-FITC (clone J.33), anti-CD11b-FITC (clone Bear1), and anti-CD66b-FITC (clone 80H3), and isotypes IgG1-FITC and PE (clone 679.1 Mc7) were from Beckman Coulter (Miami, FL, USA). For MP labeling, 30 μL of freshly thawed PFP was incubated 30 minutes with 10 μL of specific antibody or concentration-matched isotype control. Platelet microparticles (PMP) (CD41+), leukocyte microparticles (LeuMP) (CD45+), polymorphonuclear neutrophil microparticles (NeuMP) (CD66b+/CD11b+), and endothelial microparticles (EMP) (CD31+/CD41-) analyses were performed on Cytomics FC500 flow cytometer (Beckman Coulter) using a Megamix beads (BioCytex) calibrated protocol as previously described. Flow Count Fluorospheres (Beckman Coulter) were added to each sample in order to express MP counts as absolute numbers [16].

### Statistical analysis

Continuous variables were presented as mean ± SD and compared using Student's two-tailed *t*-test. Normality of the distribution for the variables was assessed using the Kolmogorov-Smirnov test. Non-normally distributed continuous variables were presented as median and interquartile range and compared using Wilcoxon's rank-sum test. The chi-square test or the Fisher exact test was used to compare categorical variables. To examine linear correlations between two variables, the Pearson or the Spearman correlation methods were used as appropriate. Multivariate logistic regression was used to identify the independent factors associated with death at Day 28. Hosmer Lemeshow test with *P *> 0.05 suggest a good fit between data and the logistic regression model. All variables that exhibited a *P*-value < 0.2 on univariate analysis were entered in the model. Interactions were tested in the model; variables strongly associated with other(s) were not included in the multivariate analysis. The following variables evaluated at Day 1 were finally entered in the model: age, SOFA score on inclusion, plateau pressure, arterial pH, circulating LeuMP. The median value of LeuMP was used as the threshold. A two-tailed *P *≤.05 was considered statistically significant. Statistics and figures were performed with SPSS 15.0 (SPSS Inc., Chicago, IL, USA).

## Results

### Patients

All patients were included within the first 48 hours of the diagnosis of ARDS according the AECC criteria. Table 1 compared the clinical characteristics of the 52 ARDS patients with the ventilated control group (VC) (*n *= 10) and with the spontaneous breathing control group (SB) (*n *= 12). As illustrated in table 1, pneumonia was the most common cause of ARDS. There were 31/52 (59.6%) ARDS survivors at Day 28. Tables 2 and 3 compared the baseline values of the biological and physiological parameters of the 52 ARDS patients according to the outcome. As expected, the non-survivors had higher SAPS II score (51 ± 11 vs. 44 ± 15, *P *= 0.05) and higher SOFA score (10 (11 to 14) vs. 7 (9 to 11), *P *= 0.007) on inclusion. Among the biological variables detailed in Table 2, only platelet count was significantly lower in nonsurvivors on inclusion. As shown in Table 3, these patients presented severe lung function impairment as reflected by the mean PaO_2_/FiO_2 _ratio, which was <120 mmHg despite a mean PEEP level of 12 cmH_2_O. Survivors and nonsurvivors did not differ in baseline respiratory and hemodynamic parameters, except that non-survivors had lower arterial pH.

**Table 1:** Clinical characteristics of the studying populations

Variables	Spontaneous breathing controls	Ventilated controls	ARDS patients	* P*-value
**Number of subjects**	12	10	52	
**Age **(years, mean ± SD)	59 ± 13	58 ± 14	58 ± 17	0.98
**Male sex **(n, %)	8 (61)	6 (60)	39 (75)	0.78
**SAPS II **(mean ± SD)	-	48 ± 13	47 ± 14	0.91
**SOFA score **(median (IQR))	-	7 (7 to 11)	10 (7 to 12)	0.11
**Admission category **(n, %)	-			0.28
**Medical**		10 (100)	42 (80)	
**Surgical**		0 (0)	10 (20)	
**Direct lung injury **(n, %)	-	-	47 (90)	-
**Cause of ARDS **(n, %)				-
**Pneumonia**	-	-	38 (73)	
**Aspiration**	-	-	6 (12)	
**Lung contusion**	-	-	3 (6)	
**Extra pulmonary sepsis**	-	-	5 (9)	
				
**Reason for ICU hospitalization **(n, %)	-		-	
**Coma**		4 (40)		-
**Self-poisoning**		3 (30)		
**CNS infections**		2 (20)		
**Epilepsy**		1 (10)		
**LISS at inclusion **(median (IQR))	-	0.75 (0.44 to 1)	3 (2.5 to 3.25)	<0.001
**Septic shock at inclusion **(n, %)	-	6 (60)	43 (83)	0.44
**Days under mechanical ventilation before inclusion **(median (IQR))	-	2 (0 to 7)	1 (0 to 1)	0.14

ARDS, acute respiratory distress syndrome; CNS, central nervous system; IQR, interquartile range; LISS, lung injury severity score; SAPS II, Simplified acute physiology score II; SD, standard deviation; SOFA score, Sequential Organ Failure Assessment score.

Septic shock was defined as a systolic blood pressure of ≤ 90 mm Hg, despite adequate volume expansion requiring the use of vasopressor agents; *P*-values compare ventilated controls and ARDS patients.

**Table 2:** Baseline biological variables of the 52 ARDS patients according to the outcome at Day 28

Variables	Survivors	Nonsurvivors	*P*-value
	(*n *= 31)	(*n *= 21)	
**Blood Neutrophils **(×10^9 ^cells/L) (mean ± SD)	11.6 ± 6.5	12.8 ± 8.0	0.6
**Hematocrit **(%) (mean ± SD)	32 ± 6	30 ± 6	0.42
**Platelet count **(×10^9 ^cells/L) (mean ± SD)	230 ± 136	154 ± 102	0.04
**Prothrombin time **(%) (mean ± SD)	65 ± 17	56 ± 20	0.09
**Fibrinogen **(g/L) (mean ± SD)	5.6 ± 1.7	4.5 ± 2.2	0.09
**Lactate **(mmol/L) (median (IQR))	1.5 (1.1 to 2.6)	1.8 (1.2 to 3.3)	0.46
**Creatinine **(μmol/L) (median (IQR))	76 (59 to 112)	108 (66 to 194)	0.08
**Procalcitonin **(ng/mL) (median (IQR))	1.2 (0 to 8.6)	4.4 (0 to 10.7)	0.44
**BAL Neutrophils **(×10^9 ^cells/L) (median (IQR))	372 (94 to 1,995)	367 (111 to 1,425)	0.67
**BALF to plasma total protein ratio **(median (IQR))	0.24 (0.14 to 0.44)	0.28 (0.16 to 0.63)	0.59

Data are expressed as mean ± SD or median and 25 to 75% interquartile range (IQR). BAL, broncho alveolar lavage.

**Table 3:** Baseline respiratory and hemodynamic parameters of the 52 ARDS patients

Variables	Survivors	Non-survivors	*P*-value
	(*n *= 31)	(*n *= 21)	
**PaO2/FiO2 **(mmHg) (mean ± SD)	114 ± 34	106 ± 33	0.4
**PaCO2 **(mmHg) (median (IQR))	43 (40 to 57)	46 (40 to 52)	0.6
**FiO2 **(mean ± SD)	0.69 ± 0.14	0.75 ± 0.18	0.2
**Total PEEP **(cmH_2_O) (mean ± SD)	11.5 ± 2.7	13.2 ± 3.4	0.7
**P plat **(cmH_2_O) (mean ± SD)	25.9 ± 6.1	27.7 ± 6.8	0.3
**qsComp **(mL.cmH_2_O^-1^) (mean ± SD)	33.8 ± 13.6	29.8 ± 10.9	0.2
**MV **(L/min) (mean ± SD)	9.5 ± 2.2	9.3 ± 2.6	0.7
**TV **(mL/Kg PBW) (median (IQR))	6.00 (6.00 to 6.20)	6.00 (6.00 to 6.05)	0.2
**HR **(beats/min) (mean ± SD)	105 ± 27	113 ± 25	0.3
**MAP **(mmHg) (mean ± SD)	74 ± 17	68 ± 14	0.2
**Arterial pH **(median (IQR))	7.34 (7.28 to 7.43)	7.26 (7.18 to 7.35)	0.01
**Vasopressor **(μg.kg^-1^.minute^-1^) (median (IQR))	0.22 (0.08 to 0.50)	0.36 (0.15 to 0.88)	0.2

FiO2, fraction of the inspired oxygen; HR, heart rate; MAP, mean arterial pressure; vasopressor was norepinephrine or epinephrine; MV, minute ventilation; PaCO2, partial pressure of carbon dioxide; PaO2/FiO2, ratio of the partial pressure of arterial oxygen and the fraction of the inspired oxygen; PBW, predicted body weight; PEEP, positive end-expiratory pressure; Pplat, plateau pressure; qsComp, quasi-static respiratory compliance; TV, tidal volume.

Results are expressed as mean ± SD, or median (IQR) as appropriate.

### Characterization of microparticles in BAL from ARDS

**Figure 1:**
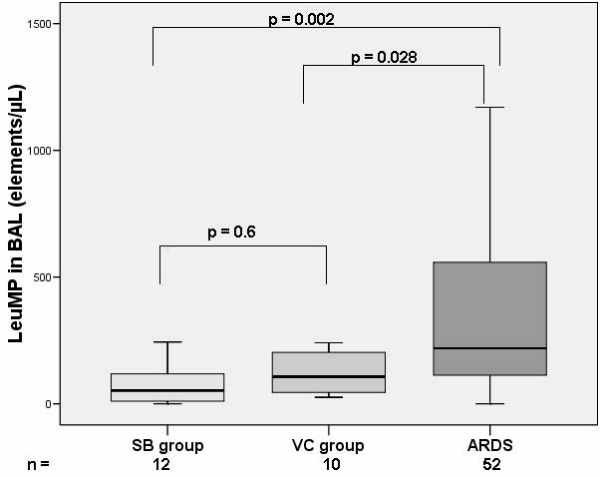
**Levels of LeuMP from BAL in ARDS patients and controls groups**. LeuMP, leukocyte microparticles; SB group, spontaneous breathing group; VC group, ventilated control group; Levels of MP are expressed as the number of elements per microliter (μL). Box plots represent median, interquartile range, 10^th ^to 90^th ^percentiles.

The analysis of the microparticles origin in BAL indicates that microparticles originating from leukocyte (LeuMP), neutrophil (NeuMP), platelet (PMP) and endothelial cells (EMP) were detected in BAL. EMP were detected in the BAL of only 6 of the 52 ARDS patients, in 1 patient in the VC group and they were not detected in the SB group. LeuMP were found higher in the BAL from ARDS patients as compared to both the VC group and the SB group (Figure 1).

**Figure 2:**
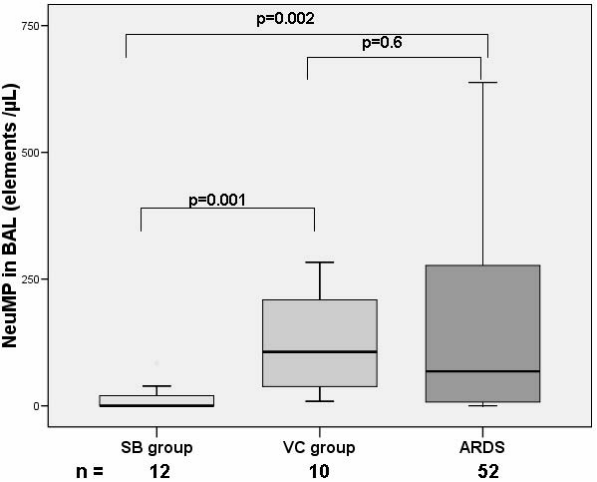
**Levels of NeuMP from BAL in ARDS patients and controls groups**. NeuMP, neutrophil microparticles; SB group, spontaneous breathing group; VC group, ventilated control group; Levels of MP are expressed as the number of elements per microliter (μL). Box plots represent median, interquartile range, 10^th ^to 90^th ^percentiles.

Similarly, NeuMP were also found higher in ARDS patients as compared with the SB group but not with the VC group (Figure 2). PMP numbers were not significantly different among the three groups (data not shown).

### Subgroup analysis

When the analysis was restricted to the ARDS related to proven bacterial pneumonia, main differences remained. We found both higher levels of LeuMP (219 (110 to 580) vs 52 (7 to 128), *P *= 0.002) and higher levels of NeuMP (68 (0 to 304), *P *= 0.002) between the ARDS related to pneumonia group and the SB group. LeuMP were also found to be higher in the ARDS related to pneumonia group as compared with the VC group (219 (110 to 580)) vs 107 (44 to 208)), *P *= 0.028 respectively).

### Microparticles at the onset of ARDS and outcome

**Figure 3:**
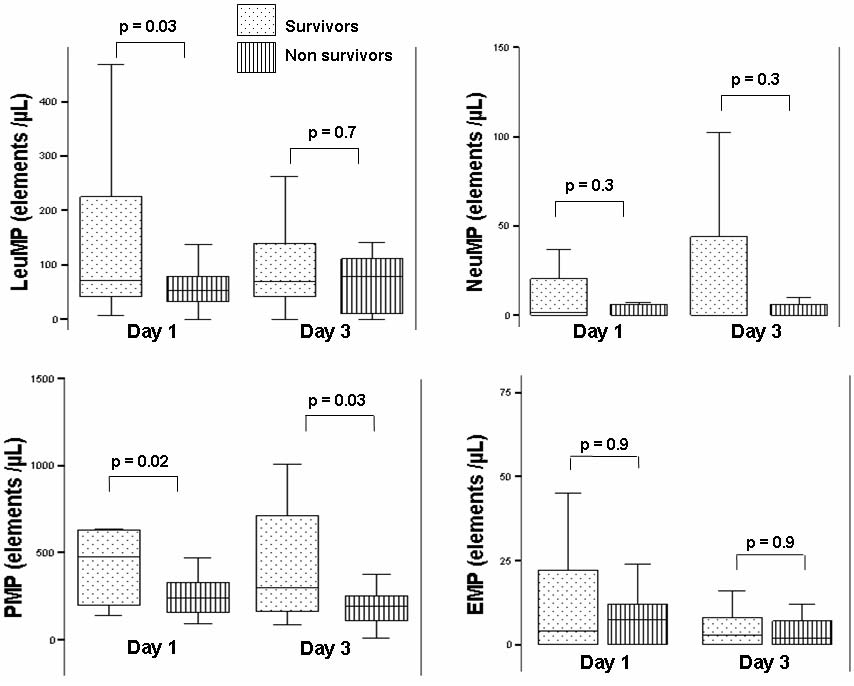
**Levels of circulating microparticles between survivors and non survivors**. LeuMP, leukocyte microparticles; NeuMP, neutrophil microparticles; PMP, platelet microparticles; EMP, endothelial microparticles. *P*-values were calculated by Wilcoxon rank-sum test. Box plots represent median, interquartile range, 10^th ^to 90^th ^percentiles.

As presented in Figure 3, we detected higher circulating LeuMP and PMP at Day 1 in the blood from survivors than in the non-survivors (*P *= 0.03 and *P *= 0.02, respectively). Moreover, three days after the onset of ARDS, PMP remained significantly higher in survivors (*P *= 0.02). Patients who had more than five VFD (mean value of the ARDS group) at Day 28 presented higher levels of circulating LeuMP (127 (53 to 273) vs 55 (28 to 115) *P *= 0.021) and circulating PMP (588 (224 to 1417) vs 257 (160 to 439), *P *= 0.014) as compared with patients who had less than five VFD. Finally, the LeuMP level in BAL performed at Day 1 was correlated with quasi-static respiratory compliance (*r*^2 ^= 0.35, *P *= 0.01).

Results of the logistic regression analysis for 28-day survival are presented in Table 4. Circulating LeuMP >60 elements/μL were associated with survival at Day 28. In contrast, severe arterial acidosis (pH <7.30) at Day 1 was associated with a worse prognosis.

**Table 4:** Factors associated with survival at 28 days

Variable	Odds ratio (95% CI)	*P*-value
**Age **(per one point increase)	1.01 (0.96 to 1.07)	0.530
**Circulating LeuMP **(<60 ^a^)	5.26 (1.10 to 24.99)	0.037
**SOFA **(>10 ^b^)	0.3 (0.06 to 1.38)	0.123
**pH **(<7.30 ^c^)	0.18 (0.03 to 0.91)	0.039
**P plat **(cmH_2_O) (per one point increase)	1.00 (0.89 to 1.12)	0.953

Hosmer-Lemeshow statistic: *P = 0.41*; 95% CI, 95% confidence interval; SOFA, Sequential Organ Failure Assessment; Circulating LeuMP, Leukocytes microparticles in plasma. ^a ^median value of circulating leukocytes microparticles at Day 1; ^b ^median value of SOFA score at Day 1; ^c ^median value of arterial pH at Day 1.

### Short term variations of microparticles during ARDS

**Figure 4:**
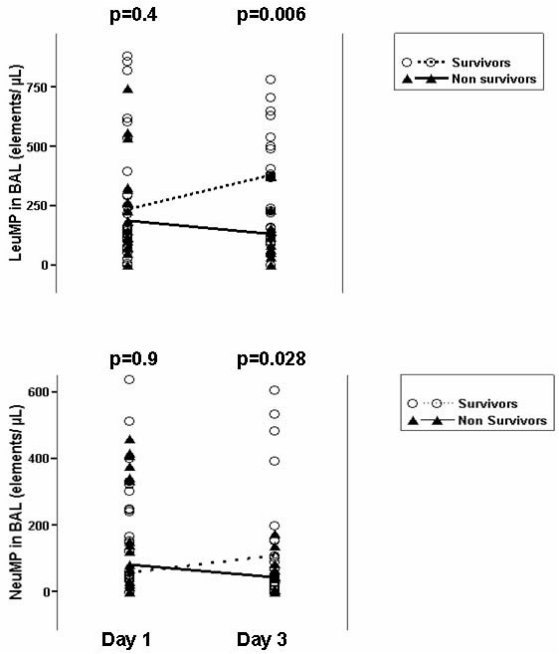
**Short terms variations of leukocyte and neutrophil microparticle in broncho-alveolar lavage**. LeuMP, leukocyte microparticles; NeuMP, neutrophil microparticles; BAL, bronchoalveolar lavage. Empty circles represent survivors and full triangles represent the non survivors. The dotted line connects the median values of MP in survivors and the solid line connects the median values of MP in non survivors. *P*-values compare survivors vs non survivors and were calculated by Wilcoxon rank-sum test.

Figure 4 shows the short term variations of leukocyte and neutrophil microparticles in the BAL from ARDS patients between Day 1 and Day 3. Survivors exhibit higher levels of those microparticles than non-survivors only at Day 3. We didn't find any difference for PMP and EMP (data not shown).

### Relation between levels of Microparticles in blood and BAL compartments

When platelet counts and PMP levels in the blood compartment were studied together, we found a significant correlation between platelet count and PMP at Day 1 (*r = 0.41, P = 0.008*). In the BAL compartment, at Day 1, LeuMP were correlated with total cellular count in BAL (r = 0.55, *P *< 0.0001) and NeuMP with total neutrophils count (r = 0.51, *P *< 0.0001). These data suggest a link between MPs levels and their parent cells in each compartment.

## Discussion

To our knowledge, this is the first study which characterized the cellular origin of MPs in the course of ARDS, both in circulating blood and BAL. The major findings are that 1/MPs originating from platelets, endothelial cells and leukocytes were detectable in the BAL of patients with ARDS 2/among them, only LeuMP and NeuMP were elevated in BAL when compared with the spontaneous breathing group 3/Higher levels of circulating LeuMP detectable early at time of diagnosis of ARDS were associated with better prognosis.

As a result of the local and systemic pro-inflammatory responses associated with ARDS, leukocytes become activated within the general or in the pulmonary circulation. In the present study, the analysis of MP subpopulation reflects the origin and the activation status of the cells present in the BAL. Accordingly the low number of granulocytes in BAL from spontaneous breathing (SB) controls is consistent with the fact that NeuMP represent a minor sub-population. The major influx of neutrophils toward the lungs early in the course of ARDS is suggested by the high levels of NeuMP recovered by BAL.

The general belief is that MPs are conveyers of deleterious information associated with exaggerated inflammatory response [8]. Indeed, elevated levels of MPs from platelets, granulocytes, and endothelium are found in patients with septic shock, meningococcemia, traumatic brain injury, and severe trauma [5-7,17,18]. In contrast, Soriano *et al. *have suggested that MPs presented protective effects in patients with septic shock [19].

Bastarache *et al. *[20] reported the presence of procoagulant microparticles in the lung from ARDS patients. By focusing on the epithelial alveolar origin of microparticles, they reported higher concentrations of these MPs in ARDS patient's edema fluid compared to patients with hydrostatic pulmonary edema. However, this latter study reported a trend for higher concentrations of total MPs in the edema fluid from non-survivors. Although this latter result did not reach statistical significance, differences with our study might be due to the cellular origin of the MPs (alveolar epithelial vs. leukocyte or platelet lineages) or to the different compartments where MPs were isolated (edema fluid vs. blood).

The main finding of the present study is that lower levels of leuMP are detectable in blood (day 1) and BAL (day 3) in non survivors.

ARDS is clinically characterized by a strong alteration of ventilator mechanics with decreased lung compliance. During ARDS, plateau pressure must be monitored and maintained as low as possible to reduce ventilator-induced lung injury or right heart failure. This can be achieved by reducing tidal volume on the ventilator and by setting an appropriate level of positive end expiratory pressure (PEEP). In previous studies, clinical parameters such as plateau pressure and quasi-static pulmonary compliance were found being strongly associated with mortality occurring during ARDS [21,22]. To assess if levels of microparticles may reflect some part of ventilatory induced lung injury, it would be interesting to search some correlation between levels of MP and the ventilatory mechanics parameters in different ventilatory conditions.

ARDS from pulmonary origin is characterized by a local pro-inflammatory process that first occurs in damaged lung and then contributes to multi-organ dysfunction. A balance between pro-inflammatory and anti-inflammatory effects is observed in the course of acute lung injury. Sustained high levels of pro-inflammatory biomarkers are associated with poor outcome of ARDS [23,24]. In the present study, the observation that low levels of MPs are associated with mortality is in agreement with data already reported for severe sepsis [19].

The initial theory for death-associated sepsis was that multiple organ failure resulted from an excessive or uncontrolled inflammatory response. A more recent concept to explain the different outcomes during sepsis is that normal responses to injury can be immunosuppressive, inducing "an immune paralysis" and leading to health care associated infections, multiple organ failure and, finally, death [9,25]. Consistent with this theory one could speculate that the lows levels of MPs in patients with worse outcome, reflect "suppression of vesiculation", given the fact that vesiculation is a response of cells to injury. Although the mechanism supporting this potential protective effect remains to be elucidated, they can be reliable to the anti-inflammatory effect of polymorphonuclear neutrophil derived MPs (NeuMP) reported by the work of Gasser and Schifferli [26]. They showed that NeuMP block the response of macrophages to LPS and increased the secretion of transforming growth factor beta1, a potent inhibitor of macrophage activation. Thus, in the earliest stage of inflammation, neutrophils cells release MP that convey potent anti-inflammatory effects, by driving the resolution of inflammation. More recently, it was reported that MPs shed from adherent neutrophils bear Annexin 1, an endogenous anti-inflammatory protein able to inhibit neutrophil adhesion to the endothelium [27]. It could have been interesting to assess some biomarkers that evaluate endothelial permeability (VWF or Ang2), the inflammation (IL-6 or IL-8) or apoptosis like FasLigand or soluble FAS.

Furthermore, our data suggesting that circulating LeuMP are protective upon ARDS onset may also be reliable to the MPs beneficial properties exerted on vascular tone during sepsis. Mostefai *et al. *[28] showed that the number of total circulating and platelet microparticles in patients with septic shock was increased and that these microparticles were protective against vascular hyporeactivity.

The mechanism supporting this protective effect in ARDS is a challenging question. One can hypothesize that blocking inflammatory cytokines such as TNF-α could suppress the release of MPs that may be beneficial in ARDS patients, which may help explain why anti-inflammatory therapies such as anti-interleukin-1R or anti-TNF have largely been unsuccessful in this context [4]. Thus, if we consider subpopulation of MP such leuMP as protector, our results support the negative impact of decreasing inflammatory response early in the course of ARDS and enlights the potential of therapeutic option aimed to promote the release of these MPs subpopulation.

Mechanical ventilation has been reported for modulate the platelet microparticles release in an experimental context [29]. Our data do not support this hypothesis to explain the differences observed between the VC group and the ARDS group concerning LeuMP.

### Limitations

The first limitation of our study is to extrapolate our results to a mixed population of ARDS. Indeed, the patients had direct lung injury in 90% with pneumonia in 73% of cases. One other limitation of our study is that circulating MPs in ARDS patients were not compared to those of the control groups. However, in contrast to MPs in BAL, low levels of circulating MPs in healthy subjects have been extensively reported in the literature [6,30] and such comparison is beyond the question raised by this study focused on the outcome of patients. This study can only provide relationship between microparticles and clinical outcome in patients with established ARDS. It was not designed to assess the association between MPs and the development of ARDS.

## Conclusions

Analysis of the cellular origins of MPs in ARDS patients identified circulating LeuMPs as a possible biomarker associated with outcome. In the future, elucidation of the mechanisms supporting the release of MPs with potential protective properties is an emerging challenge to delineate new therapeutics strategies based on physiopathology of ARDS.

## Key messages

• The leukocyte microparticles are elevated in BAL from ARDS patients.

• The circulating leukocyte microparticles are associated with prognosis during ARDS course.

## Abbreviations

AECC: American-European Consensus Conference; ALI: acute lung injury; ARDS: acute respiratory distress syndrome; BAL: bronchoalveolar lavage; EMP: endothelial microparticle; LeuMP: leukocyte microparticle; LISS: lung injury severity score; MP: microparticles; NeuMP: neutrophil microparticle; PEEP: positive end expiratory pressure; PFP: platelet-free plasma; PMP: platelet microparticle; SAPS II: Simplified Acute Physiology Score II; SB: spontaneous breathing; SOFA: Sequential Organ Failure Assessment; VC: ventilated control.

## Competing interests

The authors declare that they have no competing interests.

## Authors' contributions

CG included all the patients, performed the BAL procedures and drafted the manuscript. RL performed cytometry analysis. JMF and AR participated in the study and study analysis. CG, RL, LCJ, LP and FDG participated in the interpretation of the results and gave the advices for improving the manuscript. LP, LCJ and FDG initiated the study, participated in the design of the protocol and helped to draft the manuscript. All authors read and approved the final manuscript.
